# Effect of different concentrations of soybean lecithin and virgin coconut oil in Tris-based extender on the quality of chilled and frozen-thawed bull semen

**DOI:** 10.14202/vetworld.2017.672-678

**Published:** 2017-06-20

**Authors:** A. A. Tarig, H. Wahid, Y. Rosnina, N. Yimer, Y. M. Goh, F. H. Baiee, A. M. Khumran, H. Salman, M. A. Assi, M. Ebrahimi

**Affiliations:** 1 Department of Veterinary Clinical Studies, Faculty of Veterinary Medicine, Universiti Putra Malaysia, UPM, 43400 Serdang, Selangor Darul Ehsan, Malaysia; 2 Department of Dairy Production, Faculty of Animal Production, University of Khartoum, Sudan; 3 Department of Veterinary Pre-Clinical Sciences, Faculty of Veterinary Medicine, Universiti Putra Malaysia, UPM, 43400 Serdang, Selangor Darul Ehsan, Malaysia

**Keywords:** bull semen, cryopreservation, quality parameters, soybean lecithin, virgin coconut oil

## Abstract

**Aim:**

The objective of this study was to evaluate the effects of different concentrations of soybean lecithin (SL) and virgin coconut oil (VCO) in Tris-based extender on chilled and frozen-thawed bull semen quality parameters.

**Materials and Methods:**

A total of 24 ejaculates were collected from four bulls via an electroejaculator. Semen samples were diluted with 2% VCO in Tris-based extender which consists of various concentrations of SL (1, 1.25, 1.5, and 1.75%). A 20% egg yolk in Tris used as a positive control (C+). The diluted semen samples were divided into two fractions; one for chilling which were stored at 4°C for 24, 72, and 144 h before evaluated for semen quality parameters. The second fraction used for freezing was chilled for 3 h at 4°C, packed into 0.25 mL straws and then cryopreserved in liquid nitrogen. The samples were then evaluated after 7 and 14 days. Chilled and frozen semen samples were thawed at 37°C and assessed for general motility using computer-assisted semen analysis, viability, acrosome integrity and morphology (eosin-nigrosin stain), membrane integrity, and lipid peroxidation using thiobarbituric acid reaction test.

**Results:**

The results showed that all the quality parameters assessed were significantly (p<0.05) improved at 1.5% SL concentration in chilled semen. Treatment groups of 1, 1.25, 1.5, and 1.75% SL were higher in quality parameters than the control group (C+) in chilled semen. However, all the quality parameters in frozen-thawed semen were significantly higher in the C+ than the treated groups.

**Conclusion:**

In conclusion, supplementation of 1.5% SL in 2% VCO Tris-based extender enhanced the chilled bull semen. However, there was no marked improvement in the frozen-thawed quality parameters after treatment.

## Introduction

The procedure of cryopreservation results in low semen quality that leads to reduced conception rate. Chilling temperatures are less detrimental to the intactness of the bull semen than freezing-thawing procedure, which results in more damage to the spermatozoa [1]. In New Zealand and Australia, studies have shown the opportunity of utilization and commercialization of chilled bull semen [2]. According to Verberckmoes *et al*. [2], in New Zealand, 95% of the semen used for (artificial insemination [AI]) in cattle is processed as chilled semen. The major benefits linked to the utilization of cool-stored semen are a greater viability than frozen semen, the chance of consuming little doses for insemination, reduced expenses of storage, and the ease for utilizing in AI [3]. Moreover, chilled semen has more viability in the reproductive tract of female and guaranteeing greater rates of fertilization [4].

Egg yolk is commonly used in semen extenders; the ease of which egg yolk is affected by microbial contamination necessitates the development of alternatives to egg yolk in semen extenders. Consequently, efforts are aimed to develop chemically defined extenders, free of compounds that are of the animal sources [5]. Apart from being linked to microbial contamination, egg yolk extenders are known to interfere with the microscopic examination. Thus, replacement of such extender with plant source compound without compromising the desired qualities of perfect extender is very much warranted [6]. Thun *et al*. [7] and Aires *et al*. [8] who indicated that soybean lecithin (SL) has the same egg yolk components utilized for preventing animal semen from cold shock on frozen-thawed sperm. Moreover, according to Fukui *et al*. [9], who reported that SL might have a greater safety function for the sheep sperm than that of the egg yolk or other ingredients through cryopreservation procedure and thus, decrease the threat of introducing mycoplasma and bacteria in the frozen extenders. Singh *et al*. [1] showed that 25% soy milk based extender gave better quality parameters for bull semen at 5°C at different time interval. Zhang *et al*. [10] reported that the addition of 6% SL to the extender showed the best spermatozoa motility and plasma membrane integrity in frozen-thawed boar spermatozoa. SL is considered a better oil-in-water solvent in the candy industry [11].

Various additives such as antioxidants, fatty acids, and sugars have been added to reduce the damage of sperm through the frozen-thawed procedure [12-15]. One of these additives that is saturated and unsaturated fatty acids is contained in virgin coconut oil (VCO) [16]. Moreover, VCO has plenty of antioxidants such as tocotrienol, polyphenols, and tocopherols [17,18]. Since VCO is hydrophobic, it cannot dissolve in the extender. However, VCO can be emulsified with lecithin an excellent natural agent of emulsification [19]. To our knowledge, the use of VCO with SL in Tris-based extender and its effect on chilled and cryopreserved bull sperm has not been reported. Thus, this experiment was conducted to evaluate the effect of different concentrations of SL with 2% VCO in Tris-based extender on chilled and frozen-thawed quality parameters of bull semen.

## Materials and Methods

### Ethical approval

Approval for this study was given by the Institutional Animal Care and Use Committee of UPM with AUP No: R073/2015.

### Animals

Semen samples were collected from four mature and fertile Brangus–Simmental crossbred bulls from the Universiti Putra Malaysia (UPM) Farm. Bulls aged about 5-6 years old and weighed about 640-650 kg. The four bulls were under a similar management of feeding with *Brachiaria decumbens* grass and commercial palm kernel cake which contains about 2.6% crude fat and 16% crude protein provided at an average of 3 kg/bull/day. Mineral blocks and water were provided *ad libitum*. Semen collection and preparation of extenders

Ejaculates were collected from each bull twice per week, at 3 days interval by electroejaculation (Electrojac 6, USA). A total of 24 ejaculates were collected, and the samples were stored in a cooler box containing warm water at 37°C and transported to the laboratory for evaluations. Ejaculate samples collected with ≥80% normal sperm morphology, ≥70% general motility, and semen concentration of ≥500 × 10^6^/ml [20] were included in the experiment. The semen samples were then extended with fraction one of the Tris (2.42 g) - citric (1.48 g) - fructose (1 g) extenders [21] as the main extender with different percentage of SL (SL P5638, Sigma–Aldrich, USA) (0%, 1%, 1.25%, 1.5%, and 1.75%) containing 2% VCO (VCO; manufactured by Nano Xan Sdn. Bhd and Malaysia Agriculture Research and Development Institute, Malaysia). The fraction one contains the main extender without glycerol; it was also used for chilled treatments. The other (fraction two) besides the basic extender contains glycerol 12.8 ml for the frozen treatments. The treatment groups in chilled and frozen were divided into seven groups (Table-1). Semen sample was diluted to adjust the concentration of 20 × 10^6^ sperm in a 0.25 ml straw, subsequently gradually refrigerated to 4°C for 3 h. Straws were stuffed with diluted semen (at 4°C) and kept at the same temperature for 4 h to equilibrate. Filled straws of diluted semen were then cryopreserved in liquid nitrogen. In the freezing step, the straws were put on racks and placed horizontally 4 cm above the surface of the liquid nitrogen for 10 min. The racks were left to plunge with the straws on liquid nitrogen for 3 min before immersing them into the liquid nitrogen [20].

**Table-1:** Characteristic of diluting media used in this experiment (2% VCO+SL%).

Treatments	Components of diluting medium
Control (C+) (20% egg yolk)	Tris-based extender (Amirat-Briand *et al*., 2010)
Control (C−) (2% VCO)	Tris + 2% VCO
Treatment 1	Tris + (1% SL+2% VCO)
Treatment 2	Tris + (1.25% SL+2% VCO)
Treatment 3	Tris + (1.5% SL+2% VCO)
Treatment 4	Tris + (1.75% SL+2% VCO)

VCO=Virgin coconut oil, SL=Soybean lecithin

### Sperm motility, viability, and morphology

Sperms concentration and general motility were tested using computer-assisted semen analysis (IVOS Hamilton Thorne Biosciences, USA version 12.2). Semen sample viability and morphology were measured by eosin–nigrosin stain [20]. A smear was made from one drop (10 µl) of semen and three drops (30 µl) of the stain on a warm slide. The stained slide was viewed under a phase-contrast microscope at 400× magnification, and total of 200 sperm was calculated in an average of four microscopic fields. Sperm that did not absorb the eosin–nigrosin stain was considered alive, whereas those that absorbed the stain were dead [13]. Sperm morphology was examined by the same slide that was used for viability evaluation. The percentage of normal sperm cells was evaluated from 200 sperm cells examined.

### Plasma membrane integrity

The plasma membrane integrity was examined by hypo-osmotic swelling test as described by Kaka *et al*. [22]. 100 µl of semen were mixed with 1 µl of hypo-osmotic solution (7.35 g trisodium citrate and 13.51 g fructose dissolved in 1 L distilled water; osmolarity of 150 mOsm/kg.) and kept for 1 h at 37°C. After that, 15 µl of the solution was put on a pre-warmed slide covered with a cover slip and sperms were assessed under a phase-contrast microscope at 400× magnification. A total of 200 spermatozoa per slide were calculated from four different microscopic fields and expressed in percentage. The swelled spermatozoa were considered normal sperm cells that in response to the hypo-osmotic test solution.

### Acrosome integrity

Intact acrosome was evaluated by semen smear mixed with eosin–nigrosin stain and tested under a light microscope at 1000× magnification oil immersion as developed by Yildiz *et al*. [23]. 200 spermatozoa were examined for either intact acrosome or detached.

### Lipid peroxidation (LPO) test

Thiobarbituric acid reaction substances (TBARs) were used to measure the LPO as described by Kaka *et al*. [24]. A total of 500 µl of sperm was blended with TBARs solution and after that boiled in a water bath at 95°C for 1 h till the combination looked pink. Subsequent to cooling, 1 µl of distilled water and 3 µl of n-butanol were supplemented with the mixture and then vortexed. The combinations were centrifuged at 5000 rpm for 10 min. The absorbance of the supernatant was read against an appropriate blank at 532 nm by spectrophotometer (Secomam, Domont, France). The malondialdehyde (MDA) was calculated from a standard curve of 1, 1, 3, 3-tetraethoxypropane and expressed as nmol/3×10^8^ sperm.

### Statistical analysis

Using the PROC UNIVARIATE method, the data normality was checked, and all parameters were found to fit the normal distribution. Data on the effect of various percentages of SL on the semen quality parameters chilled or frozen-thawed in Tris-based extenders containing 2% VCO and MDA were analyzed using one-way ANOVA with the general linear model method of the SAS version 9.2. Comparisons between various percentage means were analyzed using Duncan multiple range test following significant F-test in ANOVA. All statistical procedures were performed at 95% confidence level.

## Results

The effects of different concentrations of SL in 2% VCO contained Tris-based extenders on chilled sperm quality parameters are shown in Table-2. The outcomes showed that after 24 h the general sperm motility, viability, morphology, membrane integrity, and acrosome integrity, improved with the increase in the SL concentration from 1% to 1.75%. Furthermore, all the treated groups except the negative control group (C−) (2% VCO) (which is considered less than the treatment groups and the C^+^ (20% egg yolk)) were significantly higher than the control group (C+). The results showed after 24, 72, and 144 h indicated that the treatment group with 1.5% SL was significantly higher than all other treated groups in term of general motility, morphology, viability, membrane integrity, and acrosome integrity. Moreover, there was an interaction between the treatments and time (storage period) on chilled semen in term of general motility, viability, membrane integrity, and acrosome integrity.

**Table-2:** Effect of different concentrations of SL and VCO in Tris-based extender on bull’s post-chilled sperm parameters after 24, 72, and 144 h (Mean±SEM).

Treatments	Parameters					
	
VCO% SL%	Time of chilling (h)	Motility%	Morphology%	Viability%	Membrane integrity %	Acrosome integrity%
C−	24	11.25±2.83^d^	98.54±0.45	10.00±2.76^d^	8.50±1.90^c^	61.17±1.62^c^
TA	24	76.08±1.80^b^	97.75±0.55	72.42±1.86^b^	69.75±1.43^ab^	86.79±1.14^a^
TB	24	81.00±1.58^ab^	98.00±0.42	76.00±1.70^ab^	72.08±1.45^a^	86.46±1.71^a^
TC	24	83.58±1.70^a^	97.46±0.54	79.75±1.38^a^	74.13±1.66^a^	86.67±1.45^a^
TD	24	82.42±1.09^a^	97.77±0.38	77.88±1.10^a^	72.42±0.98^a^	87.75±1.33^a^
C+	24	68.92±1.70^c^	98.25±0.33	67.04±1.61^c^	65.83±1.23^b^	77.92±0.98^b^
C−	72	10.25±2.84^c^	98.33±0.36	7.71±2.27^c^	6.67±2.29^c^	63.88±2.58^b^
TA	72	58.67±2.58^b^	98.29±0.42	56.50±2.32^b^	54.83±2.54^b^	73.25±1.82^a^
TB	72	63.50±3.18^ab^	98.17±0.33	59.29±2.42^ab^	58.71±2.73^ab^	77.50±1.76^a^
TC	72	69.25±2.38^a^	97.96±0.38	64.83±2.03^a^	62.29±2.14^a^	75.50±1.99^a^
TD	72	62.75±2.58^ab^	98.29±0.33	58.58±1.86^ab^	59.13±2.24^ab^	74.46±1.49^a^
C+	72	62.67±2.00^ab^	98.33±0.28	60.46±1.41^ab^	59.67±1.68^ab^	72.00±1.39^a^
C−	144	1.42±0.67^d^	98.71±0.40	1.79±0.62^c^	1.42±0.38^c^	58.08±1.25^c^
TA	144	31.83±1.66^abc^	98.25±0.51	27.42±0.97^ab^	27.04±1.47^ab^	64.71±1.37^a^
TB	144	33.67±1.69^ab^	98.38±0.32	27.59±1.39^ab^	28.46±1.57^ab^	63.92±1.48^ab^
TC	144	36.50±1.72^a^	98.33±0.31	30.42±2.18^a^	31.08±1.57^a^	63.00±1.20^ab^
TD	144	31.00±1.91^bc^	98.21±0.29	26.67±1.84^ab^	27.00±1.96^ab^	60.08±1.20^bc^
C+	144	28.67±1.72^c^	98.75±0.31	24.75±1.59^b^	25.00±1.77^b^	60.92±1.92^abc^
p value	Treatments	0.0001	0.3840	0.0001	0.0001	0.0001
	Time	0.0001	0.1095	0.0001	0.0001	0.0001
	Treatments*Time	0.0001	0.9949	0.0001	0.0001	0.0001

a,b,c,d Values with different superscripts within column at the same time show a significant difference at p<0.05. VCO=Virgin coconut oil, SL=Soybean lecithin, C−=0% SL with 2% VCO in Tris-based extender, TA=1% SL with 2% VCO in Tris-based extender, TB=1.25% SL with 2% VCO in Tris-based extender, TC=1.5% SL with 2% VCO in Tris-based extender, TD=1.75% SL with 2% VCO in Tris-based extender, C+=20% egg yolk in Tris-based extender, SEM=Standard error of mean

Table-3 shows the effects of the various concentrations of SL and 2% VCO on frozen-thawed sperm quality parameters. The positive control group (20% egg yolk) (C+) was better than all treatment groups and negative control (C−) for all quality parameters. Moreover, there were no differences between 7 and 14 days (storage period) in term of quality parameters in frozen-thawed semen. Furthermore, there was no interaction between the treatment and time of storage period on the frozen-thawed semen.

**Table-3:** Effect of different concentrations of SL and VCO in Tris-based extender on bull’s post thawed sperm parameters after 7 and 14 days (mean±SEM).

Treatments	Parameters					
	
VCO% SL%	Time of freezing (days)	Motility %	Morphology %	Viability %	Membrane integrity %	Acrosome integrity %
C−	7	3.17±1.22^b^	98.25±0.29	9.29±1.36^c^	2.83±1.03^b^	63.88±1.41^b^
TA	7	13.92±4.48^b^	97.17±0.69	28.83±5.96^b^	12.88±4.07^b^	69.54±1.40^b^
TB	7	13.92±4.51^b^	97.54±0.39	29.54±5.59^b^	12.71±4.18^b^	68.63±2.02^b^
TC	7	10.00±4.01^b^	97.96±0.50	23.83±6.27^b^	8.92±3.67^b^	67.88±2.03^b^
TD	7	9.33±3.04^b^	97.92±0.46	22.54±5.35^bc^	7.88±2.58^b^	63.08±5.86^b^
C+	7	51.17±2.85^a^	98.29±0.32	56.04±1.98^a^	50.25±2.71^a^	85.63±1.32^a^
C−	14	2.67±0.98^c^	98.50±0.28	8.42±0.82^c^	2.46±0.82^b^	65.58±1.56^b^
TA	14	13.25±4.14^b^	97.78±0.52	28.46±5.60^b^	12.00±3.71^b^	68.63±2.02^b^
TB	14	12.50±4.24^bc^	98.00±0.32	27.71±5.09^b^	11.92±3.98^b^	67.83±2.35^b^
TC	14	9.00±3.86^bc^	97.96±0.50	21.88±5.96^b^	8.33±3.48^b^	68.21±2.24^b^
TD	14	8.67±2.87^bc^	98.08±0.39	19.17±4.69^bc^	9.79±4.37^b^	67.75±1.62^b^
C+	14	50.33±2.79^a^	98.46±0.26	55.79±2.27^a^	50.17±2.63^a^	84.46±1.08^a^
p value	Treatments	0.0001	0.2600	0.0001	0.0001	0.0001
	Time	0.6716	0.1617	0.5932	0.9449	0.6590
	Treatments*Time	1.0000	0.9880	0.9995	0.9984	0.8226

a,b,c Values with different superscripts within column at the same time show a significant difference at p<0.05. VCO=Virgin coconut oil, SL=Soybean lecithin, C−=0% SL with 2% VCO in Tris-based extender, TA=1% SL with 2% VCO in Tris-based extender, TB=1.25% SL with 2% VCO in Tris-based extender, TC=1.5% SL with 2% VCO in Tris-based extender, TD=1.75% SL with 2% VCO in Tris-based extender, C+=20% egg yolk in Tris-based extender, SEM=Standard error of mean

**Figure-1:**
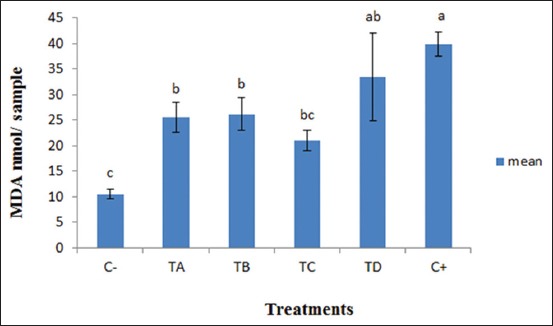
Malondialdehyde level in different concentrations of soybean lecithin (SL) % + 2% virgin coconut oil (VCO) assessed by thiobarbituric acid reaction test (mean±standard error of mean). ^a,b,c^Denote significant differences (p<0.05), error bar=SEM. MDA=Malondialdehyde, C−=0% SL with 2% VCO in Tris-based extender, TA=1% SL with 2% VCO in Tris-based extender, TB=1.25% SL with 2% VCO in Tris-based extender, TC=1.5% SL with 2% VCO in Tris-based extender, TD=1.75% SL with 2% VCO in Tris-based extender, C+=20% egg yolk in Tris-based extender.

LPO measured based on the MDA produced is shown in Figure-1. The graph showed that the MDA production was increased in treated groups of SL% with 2% VCO and it was higher in the positive control group (20% egg yolk). However, the production of MDA was decreased in the negative control group (0% SL with 2% VCO) (C−).

## Discussion

In this study, the addition of different concentrations of SL with 2% VCO in Tris-based extender showed positive effects on the quality of chilled semen parameters such as motility, morphology, viability, membrane integrity, and acrosome integrity. Furthermore, the results showed that after 24, 72, and 144 h the treatment group of 1.5% SL with 2% VCO in Tris-based extender was significantly higher in the quality parameters of chilled semen than all other treated groups and the C+ (20% egg yolk). This result coincides with earlier study which confirmed that the addition of soybean milk in the diluted buffalo semen enhanced chilled semen characteristics [1]. According to De Paz *et al*. [25], who reported that the SL extender could improve the motility and viability of chilled ram semen in 15 and 5°C which was equally effective to egg yolk extender. Akhter *et al*. [26] indicated that the semen motility and viability of buffalo sperms chilled at 5°C in SL based extender were better than in other with respect to extender such as milk, Tris-citric egg yolk, and egg yolk-citrate extender. Moreover, Rehman *et al*. [27] reported that 25% soy milk could be utilized as a substitute of traditional egg yolk-based extender for bovine sperm stored at 4°C. It is important to mention that the mixture of 1.5% SL with 2% VCO (in Tris--based extender) contains phospholipids, different types of the fatty acids, and antioxidants could play a great role to maintain the structure of sperm plasma membrane against cold shock and thus, improved the quality parameters of the sperm. Zhang *et al*. [10] explained that the better enhancement of semen parameters in SL supplemented extender is the result of its little viscosity and a lesser amount of debris. The phospholipids in SL may be replaced the phospholipids of the sperm membrane and as result of that the structure and function of sperm are maintained [28,29]. Furthermore, the phospholipids from soybean along with these of the sperm membrane would form a protective membrane against the harmful elements [10].

In this study, the supplementation of different concentrations of SL with 2% VCO in Tris-based semen extender did not improve the quality of frozen-thawed semen parameter. In contrast, the group (20% egg yolk) (C+) improved motility, morphology, viability, membrane integrity, and acrosome integrity of the frozen-thawed semen. This finding supports a previous study by Crespilho *et al*. [5], who reported that the soy lecithin represents a substitute for the growth of chemically defined extenders with reduced hygienic risk of contamination. However, egg yolk based extenders are more useful on the cryopreservation of bull post-thaw semen viability and fertility. Furthermore, De Leeuw *et al*. [30] reported that bovine spermatozoa survive freezing more efficiently in egg yolk-containing extenders than in SL. Frozen-thawed semen recovery was significantly better when the sperm was cryopreserved in egg yolk-based extender compared to lecithin-based extender. The beneficial outcome of egg yolk may be related to its cryoprotective abilities and nutritive properties [31]. On the other hand, SL lost its emulsification properties as the sperm is frozen. This is because SL is insoluble in water solutions and creates emulsions. This property of SL is a proven obstacle in the use of such extenders after hours or days of storage at 5°C and 15°C, which is essential in the field when chilled semen is used in artificial insemination. The conditions of the temperature, procedures of preparation, and time of storage may affect the formation of emulsions and availability of phospholipids in the extender [25]. Moreover, it was observed decrease of phospholipids whenever extenders reach storage temperatures of between 15°C and 5°C after a few hours. This is clarified by increased the viscosity of the extender which thought to be induced by the low temperature [32]. Moreover, Crespilho *et al*. [33] and Papa *et al*. [34] reported that the protective efficiency of SL during the freezing process of semen is limited which resulted in reduction in bovine frozen-thawed sperm survivability and fertility when compared with egg yolk-based extenders. Furthermore, according to van Wagtendonk-de Leeuw *et al*. [35], it has been reported that the influence of SL on sperm motility results from the extender viscosity and the presence of particulate debris. In addition, Üstüner *et al*. [36] suggested that the frozen-thawed method damagingly affected sperm motility and acrosome integrity.

The present results show that the MDA level in the extender of different concentrations of SL with 2% VCO in Tris-based extender was significantly lower than the positive group containing 20% egg yolk (C+). These findings substantiate results of previous studies, which showed that the components of extender effect the cryopreserved semen lipid peroxidation (LPO) [37,38]. It appears that in the present study, 2% VCO with the different concentrations of SL (1%, 1.25%, 1.5%, and 1.75%) protected sperm positively from LPO when compared to 20% of egg yolk (C+). The reason is that the structure of egg yolk contains more unsaturated fatty acid susceptible to LPO. However, this study incongruent with that of Çoyan *et al*. [39] and Atessahin *et al*. [40] who reported that sperm LPO is not influenced freezing extender. The lowest level of MDA was recorded in the (C-) (2% VCO) group. This group is produced low amount of MDA because it contained only 2% VCO (it has plenty of different types of antioxidants) and free from any type of cryoprotectants and that was the reason of low sperm parameters in this group.

## Conclusion

This study indicated that the supplementation of different concentration of SL in Tris-based semen extender (containing 2% VCO) enhanced quality parameters of chilled bull semen. Moreover, the result also demonstrated that optimal SL concentration in the extended medium was 1.5% for chilled bull semen. The Tris-based extender containing 2% VCO with different concentrations of SL% did not improve the quality parameters of frozen-thawed bull semen. Further investigations are required to enhance the freezing process using a better solvent that may improve homogenization of VCO with SL combination in extenders.

## Authors’ Contributions

AAT, HW, and YMG were designed all steps of the study. AAT, FHB, HS, AMK, and MAA have collected samples of the semen and evaluated throughout the study. AAT and ME have analyzed the data statistically. AAT, HW, YR, NY, and YMG have prepared the tables of results and discussed it. AAT wrote the manuscript draft, and all authors have read, revised, and approved the final manuscript.
